# Are owners chalk and cheese in the context of dividend smoothing asymmetry?

**DOI:** 10.3389/fpsyg.2022.969782

**Published:** 2022-10-18

**Authors:** Zahid Ali, Yang Hanming, Wu Zhongxin, Shuaib Ali

**Affiliations:** ^1^School of Accounting, Zhongnan University of Economics and Law, Wuhan, China; ^2^Department of Commerce and Management Sciences, University of Malakand, Chakdara, Pakistan; ^3^School of Management, Hainan University, Haikou, China

**Keywords:** asymmetry, dividend smoothing, ownership structure, PCA, Pakistan

## Abstract

The study analyzes the impact of ownership structure on dividend smoothing *via* the lens of agency and information asymmetry theory. The study also investigates the impact of ownership on dividend smoothing in the unexamined asymmetric context Dividend smoothing is measured *via* speed of adjustment and relative volatility. The study documents that higher individual, management, and institutional ownerships are positively associated with increased dividend smoothing. Consistent with the rental hypothesis in foreign-owned firms smooth less also concentrated firms bear with cuts and omissions. Foreign ownership has the opposite impact on dividend smoothing in adjusting dividends from below and above i.e., always prefer high dividends. Individual ownership has also exhibited a different impact in smoothing from below and above. Institutional owners avoid cuts and omissions and negatively affect SOA (smooth more) in case of adjusting dividends from above. Ownership concentration is negatively associated with dividend smoothing irrespective of whether the firm is smoothing from above or below. In contrast, management ownership negatively affected SOA in adjusting from above or below. Family firms in Pakistan smooth more to win minor shareholders' trust and signal that they sacrifice their private benefits to reduce the type II agency problem. Finally, the authors found a negative association between dividend smoothing and corporate governance quality. Over all the findings of the current study provides insight to the investors and regulators by offering dividend smoothing as an alternative monitoring mechanism to corporate governance.

## Introduction

Firms' dividend is one of the shareholder returns over their investment. The dividend announced by the company increases the share price (Gordon, 1963). However, if the company announces a dividend increase by compromising future investment projects, the market reacts negatively (Litzenberger and Ramaswamy, 1982). Many scholars have found evidence of increased prices in response to dividend initiation and adverse reactions to dividend omission and cuts (Lintner, 1956). This situation is so tricky that even managers go for external financing and forgive economically attractive projects to avoid dividend cuts (Gordon, 1963; Brav et al., 2005).

Hence, managers consider it while announcing dividend increases. The market puts a premium over relatively stable or consistently increasing dividends. Therefore, management efforts achieve this smoothness of dividends (Lintner, 1956). Most corporations have pre-defined targeted payout ratios and adjust their dividends gradually for any permanent increase in earnings.

The current study tests the impact of ownership structure on dividend smoothing through the channels of agency and information asymmetry while using data of 255non-financial firms listed on the Pakistan stock exchange (PSX) for the period of 2005-15. The study aimed at investigating the impact of ownership on dividend smoothing in the asymmetric context i.e., do owners influence dividend smoothing in the same fashion when smoothing from below or above the target payout level?

The findings of the current study provide both researchers and practitioners with new insights by investigating the role of owner types in payout decisions. It explores the role of different owners types in smoothing dividends toward the target payout ratio from above and below. These findings may assist managers, investors, and board members regarding payout decisions. Regulators prefer sustainable foreign investment. in this context rent extract behavior *via* dividend smoothing of the foreign shareholders needs their attention. Similarly, SECP and PSX want to insure minority protection, the current findings reflect that dividend smoothing is another monitoring mechanism. Over all the findings of the current study provides insight to the investors and regulators by offering dividend smoothing as an alternative monitoring mechanism to corporate governance.

This study will contribute to the limited literature on dividends smoothing; few studies have addressed the cross-sectional differences in the dividends smoothing behavior among the firms by giving attention to the firm's level financial factors and some country-level macro variables. However, minimal literature describes the ownership structure's role in the firms' dividends smoothing behavior. This study aims to fill this gap by providing empirical support as to why firms with different ownership structures go for a different level of dividend smoothing.

Most existing studies confines are exploring determinants of dividend smoothing in Pakistan. Few studies have addressed ownership association with dividend smoothing but have mostly confined to ownership concentration, institutional and family ownership e.g., Leary and Michaely (2011) ownership concentration, Du et al. (2021) individual and institutional while Ahmed et al. (2020) have addressed family ownership. In contrast, on dividend smoothing. In contrast, the current study has investigated institutional, individual, foreign, management, and concentrated ownership. The purpose of the study is to empirically analyze the impact of ownership structure on dividend smoothing for the non-financial firms listed on the Pakistan stock exchange for 2005–2015. Market responses to increases, cuts, initiations, and omissions are not symmetric. Asymmetric shock absorption means that positive and negative changes are not absorbed equally (Lintner, 1956). Further Leary and Michaely (2011), found that firms smooth more if they are smoothing from above and less otherwise. Keeping this in consideration authors answer whether the impact of ownership is symmetric i.e., using an agency or information asymmetry channel or they have an asymmetric impact in smoothing from above and below. The study has analyzed the asymmetric behavior of different ownership structures on dividend smoothing and answered whether owners like chalk and cheese when they are smoothing dividends from below and above the target payout ratio. Tobit regression is used for analyzing the impact of ownership structure on dividend smoothing measured *via* speed of adjustment, while OLS regression is used for relative volatility. The study developed the ownership index *via* principal component analysis (PCA).

The study finds individual and management ownership positively associated with dividend smoothing. The study reports that firms with higher institutional ownership exhibit higher smoothing in Pakistan. Besides this, foreign and individual ownership has the opposite impact on SOA in adjusting dividends from below and above, i.e., foreign and individual owners are chalk and cheese; when smoothing from below and above the target ratio, however, they prefer high dividends in any case. Institutional owners avoid cuts and omissions and negatively impact SOA when firms are adjusting from above. Ownership concentration negatively affects dividend smoothing irrespective of whether the firm is smoothing from above or below. In contrast, management ownership negatively impacts SOA from both directions, whether adjusting from above or below.

The remaining part of the paper is organized as follows. Section Relative literature and hypotheses development shows a review of the related literature and hypothesis development. Section Methodology of the study covers the data and research methodology used to examine the relationship between ownership structure and dividend smoothing. Section Results and discussion provides a discussion of the results. Section Conclusion shows the conclusion, including limitations, policy implications, and future directions.

## Relative literature and hypotheses development

Dividend smoothing is evident in finance literature continuously (e.g., Leary and Michaely, 2011; Lambrecht and Myers, 2012; Farre-Mensa et al., 2014; Baker et al., 2016; Wu, 2018; Garcia-Feijoo et al., 2021). Investors and market reaction are one of the reasons why firms' smooth dividends as the market put premiums over dividend initiation and increases and react negatively to omissions and cuts (Mantripragada, 1976; Gugler and Yurtoglu, 2003; Guttman et al., 2010). Lambrecht and Myers (2012) and Baker et al. (2016) have found investors' conservatism and risk aversive attitude to be the determinants of dividend smoothing. Others have associated dividend smoothing to be associated with firm, industry, and market characteristics (Leary and Michaely, 2011; Michaely and Roberts, 2012). Therefore, management puts efforts to achieve smoothness of dividends (Lintner, 1956).

Firms observe shocks of income *via* variation in debt through repayment/borrowing or *via* varying cash levels for minimizing variations in dividends. So smoothing dividends *via* investment and financing channels is evident in literature (Balli et al., 2022).

Theories relevant to dividend smoothing are related to agency conflict, information asymmetry, external financial constraints, reputation, managerial career concern, and support for risk-shifting.

Different authors have presented information asymmetry models Kumar (1988), and Guttman et al. (2010). In these models, dividends convey inside information about the current and future cash flows. Existed information asymmetry literature depicts that dividend smoothing is positively linked with equity risk factors (Kumar and Lee, 2001) and cash flow volatility (Kumar, 1988). Guttman et al. (2010) found that dividend smoothing increases as the investment horizon decreases and also increases with improvement in investment opportunities. Based on these models dividend smoothing exists because of information asymmetry therefore it should be prevailing more in the firms where the benefit of smoothing dividends is large (Manos et al., 2012; Michaely and Roberts, 2012; Balli et al., 2022). Hence young, firms with more intangible and more growth opportunities should opt for more dividend smoothing. Dividend smoothing is expected to decrease with the passage of time as long as more market analyst follows the firms and more sophisticated technology is launched. Dividend smoothing is the result of information asymmetry between the managers and owners (DeMarzo and Sannikov, 2008).

This information asymmetry between managers and owners also induces managers to smooth dividends in comparison to the earning streams for avoiding the risk of firing. (Fudenberg and Tirole, 1995). The principals develop expectations from the firm based on the current cash flows, but at the same time agents are allowed to maintain the optimal level of cash inside for safety margin in order to avoid liquidation. Therefore, the periods where performance is high, some of the shocks are observed by increasing the optimal level of cash (DeMarzo and Sannikov, 2008). So this channel is the outcome of the managers' career concern (Wu, 2018). Al-Najjar and Kilincarslan (2016) investigated the impact of family ownership, foreign ownership, domestic financial institutions and state ownership on dividends. Csabay and Stehlikova (2020) argues that Ownership structure varies with the information asymmetry (size) of the firm and the same is with dividend smoothing so it would be interesting to investigate the impact of ownership structure on dividend smoothing *via* information asymmetry channel.

It is also evident that dividend smoothing is linked with the firm behavior of smoothing i.e., low smoothing firms smooth less despite facing high information asymmetry. While high smoothing firms keep on smoothing more despite variations in information and agency conflict (Syed et al., 2018). In credit crunch periods of financial crises, firms put efforts to avoid steady and high dividend commitments to not expose themselves to costly external finances through dividend smoothing, which could be the outcome of external financial constraints (Rhee and Park, 2018). Firms operating in investor-protected environments, with low ownership concentration, and under greater regulatory pressure exhibit high dividend smoothing. Similarly, firms relying more on equity issuance smooth more for improving their access to the equity market (Koussis and Makrominas, 2019).

Agency based view of dividend smoothing reflects it as an outcome of agency cost of free cash flows. Institutional investors have efficient monitoring abilities and are valued for this. Once they invest in firms, they impose significant fines for dividend reductions and omissions (Allen et al., 2000), forcing management to implement dividend smoothing. Additionally, according to Easterbrook (1984) and Jensen (1986), providing consistent, stable dividends exposes a firm to outside financial markets, which ultimately lowers agency costs (DeAngelo and DeAngelo, 2007). High leverage improves agency expenses while reducing financial flexibility. Large and frequent payouts lower agency costs without sacrificing access to external capital at a cheap cost. To lower the high costs of free cash flows, dividend smoothing is always accompanied by sizable and frequent dividend payments in agency-based models.

Dividend smoothing should be affected by improved governance since it reduces agency conflicts and information asymmetry (Krishnamurti et al., 2005; Aggarwal and Kyaw, 2010; Zhou et al., 2013). According to Javakhadze et al. (2014). Firms in the developed world smooth more than their counterparts (cross listing 2022). Lack of transparency and weak shareholder protection in emerging markets promote the role of dividends as a signaling mechanism; hence, managers are significantly concerned about large changes in dividend payouts.

The degree of dividend smoothing changes significantly when ownership is taken into account (Fernau and Hirsch, 2019). Dividend smoothing decreases with ownership concentration in a strong governance environment where concentrated owners cannot affect minors (Syed et al., 2018; Du et al., 2021). Dividend smoothing and intuitional ownership are further alternative monitoring methods for managing agency costs (Allen et al., 2000). They could complement or substitute each other (La Porta et al., 2000). When shareholder rights are minimal, dividends are large and stable. According to Allen et al. (2000), institutional investors drive management to large and steady dividends, so they are appreciated by others. Individual investors frequently lack information and want dividends to make up for this informational insufficiency (Brennan and Thakor, 1990). Similarly, Du et al. (2021) argue that mixed ownership—majority individual, minority institutional—can be advantageous. This is because it combines access to new resources and competencies that institutional ownership may provide with the efficient monitoring and adaptable management that come with individual ownership. Such organizations may smooth less, taking into account the information asymmetry and agency view.

Dominating ownership by individual proprietors becomes less significant in established family businesses as compared to new entrants (Du et al., 2021). Through the agency channel, management social capital improves dividend smoothing while through the information asymmetry channel it reduces dividend smoothing as it improves access to external finances (institutional investment) and reduces managerial concern (Du et al., 2021). Also, Lambrecht and Myers (2012) came to the conclusion that managers' rent-seeking behavior causes dividend smoothing. They made the argument that shareholders prefer consistent dividend payments to offset rising agency costs. In addition, because they are risk averse, it also enables managers to extract rents. In a similar vein, Leary and Michaely (2011) claimed dividend smoothing, to be the result of a manager's persistent behavior.

Since 1956, dividend literature has frequently discussed dividend smoothing, although market responses to increases, cuts, and initiations and omissions are not symmetric. Asymmetric shock absorption means that positive and negative changes are not absorbed equally (Flyers for 2019). In line with Leary and Michaely (2011), this study looks into how ownership structure affects dividend smoothing in this asymmetrical context.

### Ownership concentration and dividend smoothing

Firms opt for the smoothed dividend to avoid agency conflicts. Firms facing a high conflict of interest will pay high and stable dividends in order to minimize these conflicts. High and stable dividends decrease free cash flows and ultimately ask for external financing. Following such practices, companies are compelled to follow the disciplinary forces of financial markets. Thereby leading to agency cost reduction and the arise the need to smooth dividends more. Shleifer and Vishny (1997) argue about type-II agency problem i.e., expropriation of minority shareholders by large controlling block holders.

Controlling shareholders closely monitor management but may use firm resources for their own personal benefits. Concentrated ownership firms are associated with less dividend smoothing and care less about the reduction of agency conflicts. Large block holders may bear dividend cuts as they care more about the survival of the firm. Such firms face a low level of Type-I agency conflict as they have more power to control management (Shleifer and Vishny, 1986). However, such firms have high chance of the expropriation of minority shareholders (Shleifer and Vishny, 1997).

The role of ownership structure in dividend smoothing is quite significant (Michaely and Roberts, 2012). The firm will practice less dividend smoothing relative to its earnings if it is controlled by a limited chunk of block-holders as they are less concerned with agency and asymmetric behaviors of the firm. Artikis et al. (2011) found that firms having highly concentrated ownership pay less amount of the dividend and are less likely inclined to raise it proportionately with earnings or with a decrease in debt level.

Likewise, results were obtained by Khan (2006) ascertaining the negative association of ownership concentration with dividend payout in UK firms. In order to practice opportunistic behavior, the firm's large controlling shareholders prefer to have few independent directors on the board and on audit committees. Family block holders like to have a limited number of independent directors on the board (Anderson and Reeb, 2004; Setia-Atmaja et al., 2009).

Based on the above deliberations the following hypothesis has been proposed:

***H***_**1**_***:*** ***Companies having concentrated ownership tends to smooth dividends less**.*

### Individual ownership and dividend smoothing

Individuals prefer capital gains to dividends because of the different tax treatments of capital gains and dividends (Miller and Modigliani, 1961). In Pakistan, capital gains were tax exempted before 2010, while dividends were taxed at the rate of 10% for individuals. Therefore individual ownership is negatively linked with the dividends in Pakistan (Khan, 2006; Ahmad and Javid, 2010; Afza and Mirza, 2011; Asghar et al., 2011). This discriminatory tax structure induces individual owners to prefer capital gains. While according to the behavioral model individuals consume dividends but save capital gain (Shefrin and Statman, 1984). This view is supported by Baker and Wurgler (2004), who found that individuals prefer small and smooth dividends as long as they have desired savings. Ultimately, they find a positive association of dividend smoothing with individual ownership.

However, this individual owner's preference is also affected by the information asymmetry between informed and uninformed insider individuals (Brennan and Thakor, 1990). In case of low information asymmetry, individual investors insiders who control firms will bear with small dividends and will be ready to afford dividend cuts and omissions because they will be more concerned with the long-term existence of the firm (Fairchild et al., 2014). While individuals who are facing more informed investors like institutes will ask for large dividends to reduce their Information asymmetry. In line with the above, the study hypothesizes that:

***H***_**2**_***:*** ***Individual ownership is positively associated with dividend smoothing**.*

### Foreign ownership and dividend smoothing

Literature depicts a hazy view of the association between foreign ownership and dividends.

Lin and Shiu (2003) reported that in Thailand, foreign shareholders invest in companies with low dividend yields. While this association between foreign ownership and dividends was reported positive for Korea (Jeong, 2011) i.e., foreign investors in Korea have a preference for high dividends. However, Kowalewski et al. (2008) doesn't find any significant association between foreign ownership and dividend. Liljeblom and Maury (2016) reported that foreign ownership is associated with low dividends for Russian firms. However, this result could be because of the discriminatory tax treatment of foreigners. The author reports that in Russia, dividends are taxed at the rate of 6% for locals and at 15% in the case of foreigners. Baba (2009) reported that an increase in foreign ownership of Japanese firms results increase in both the probability of dividends and the level of dividends similarly the author further proceeds and reports that an increase in foreign ownership lowers the probability of dividends reduction.

Therefore, consistent with the rental hypothesis it is hypothesized that dividends will not stick to the previous level but will be frequently changed with variation in earnings as these firms are less constrained by financial resources, further as foreign ownership is considered to be an efficient monitor so less severe agency conflict will be faced by such firms. Therefore, based on agency theory, Hence study expects that:

***H***_**3**_***:*** ***Foreign ownership and dividend smoothing are negatively associated**.*

### Institutional ownership and dividend smoothing

Institutional investors play a vital role in the capital markets because of their significant stakes in the investee firm. Being large shareholders, they are able to mitigate the agency problems between the principals and agents (Gillan and Starks, 2003). They are specialized with better know-how pertaining to firm-specific information (Edmans, 2009) and are professional investors to evaluate firms' performance, quality of management, and governance in a better way (Crane et al., 2016).

Institutional directors are independent of managers to protect shareholder's interests, hence ultimately reducing agency issues between the shareholders and managers (El-Masry et al., 2008; Colpan and Yoshikawa, 2012). Institutional investors discipline managers because of the abundance of resources and expertise in the affairs of the firm. They enhance corporate transparency and reduce practices of fraudulent accounting (Wan-Hussin, 2009). Literature depicts that institutional investors use dividend policy as a tool to extract resources from the firm. Other proponents report a positive relationship between institutional investors and dividend payout (Hovakimian and Li, 2010; Van Pelt, 2013).

Dividend smoothing controls agency cost of free cash flow. As institutional investors are important because of their strong monitoring abilities (Berger et al., 2000), therefore, managers use dividends to attract this type of investor. Once institutional investors are lured to the corporation, they force managers to avoid dividend cuts and smooth dividends. In the light of the aforementioned saying study postulates that,

***H***_**4**_***:*** ***Companies having more institutional investors will smooth dividends more**.*

### Management ownership and dividend smoothing

Proportionate ownership of directors, their spouses, and their children represent management ownership. Because of greater alignment between owners and managers, such firms have better corporate governance, therefore in support of the outcome hypothesis, such firms pay high dividends (Aoki, 2014). Directors have a propensity to pay themselves dividends (Bradford et al., 2013), but they are countered by the presence of independent directors (McGuinness et al., 2015). Also, the presence of large block holders asks for more retention and their presence reduces the need of using dividends as an alternative monitoring mechanism (Aoki, 2014). Also, CEOs with more equity ownership especially in the case of entrenched CEOs (CEO also act as chairperson, have long tenure, can influence boards, and with more ownership) pays low dividends (Ghosh and Sirmans, 2006).

In contrast, firms with low management ownership are exposed to severe agency conflict which is reduced *via* an alternative monitoring mechanism of paying high dividends (La Porta et al., 2000). Also, this view is supported by signaling theory as the cost of false signaling is high for companies with CEO's and management ownership. Keeping in view the rent extraction of managers and the countering efforts of director, managers often go for low cash but high stock dividends (Lin et al., 2010). The cost of false signaling is high and hence such companies adopt a smooth payout policy. Keeping in view the underlying mechanism of signaling theory, it is postured that more shares owned by the management, the greater is the advantage of signals. Hence, managers are expected to follow rent extraction behavior *via* opting higher level of dividend smoothing:

***H***_**5**_***:*** ***Companies with high management ownership will smooth dividends more**.*

## Methodology

### Data and sample

All listed non-financial firms on the Pakistan stock exchange (PSX) for the period of 2005–15 comprise a sample. Dividend-related data was used from 1999 to 2015. Financial firms are excluded because of their different regulatory requirements. Sample retained firms having at least 3 years' consecutive data to check smoothing patterns. Further firms not having ownership data and firms having <5 years financial data were also excluded. ultimately sample comprises of 2,744 firm-year observations for 255 non-financial listed firms classified in 12 industries. The sample size is well above the related studies in the research area e.g., 2,659 and Durana et al. (2022a). Further following Leary and Michaely (2011) two steps procedure was followed for measuring dividend smoothing which reduces small sample biasness.

EPS and DPS data for dividend smoothing measures, as well as data on controls, are acquired from the balance sheet analysis (BSA) of State Bank of Pakistan (SBP). Stock price information has been collected from www.khistocks.com and www.brecorder.com. Data on the firm's measure of risk (Beta) is collected from *open doors.pk*. Ownership structure-related data is hand collected from the annual reports of the respective companies variables descriptions is reported in Table 1.

**Table 1:** Variables description.

**Variable**	**Description**	**Relation**
**Dependent variables:**		
Speed of adjustment (SOA)	Estimated *via* equation (2)	
Relative volatility	Estimated *via* equation (3) and equation (4)	
**Control variables:**		
Firm size (SIZE)	Natural logarithm of total assets	Positive
History	The number of years Since Incorporated.	Negative
Risk (Beta)	Firm's beta at the year-end	Positive
Leverage	Total debt divided by total assets of the firm	Negative
Tangibility	Tangible Assets divided by total assets	Negative
Cash flows	Operating cash flows	Positive
**Independent variables**		
Ownership concentration (Top5_Own)	No of shares held by the five largest shareholders in proportion to the total outstanding shares.	Negative
Foreign ownership (FORGN)	No of shares held by foreign shareholders and Pakistanis residing abroad in proportion to the total outstanding shares.	Negative
Institutional ownership (INST)	No of shares are held by institutional shareholders in proportion to the total outstanding shares.	Positive
Management ownership (MGT)	No of shares held by directors, their spouses, and children in proportion to the total outstanding shares.	Positive
Individual ownership (Indiv):	No of shares are held by individuals in proportion to the total outstanding shares.	Positive

Source: Author calculations (2017).

## Variables measurement

### Dividend smoothing

Following Leary and Michaely (2011) two measures of dividend smoothing, are used i.e., speed of adjustment (SOA) and relative volatility (Rel_Vol). Following Fama and Babiak (1968) adjustments to Lintner (1956) the study used;


(1) $$\begin{array}{l} \text{Δ}{\text{D}}_{it}={\text{β}}_{0}+{\text{β}}_{1}\;EPS_{it}+{\text{β}}_{2}\;DPS_{i,t-1}+{\text{ε}}_{it} \end{array}$$

In the above equation β_2_ speed of adjustment (SOA), theoretically, SOA ranges between 0 and 1. Dividend smoothing lowers as SOA approaches 1, while SOA near-zero reveals a higher level of dividend smoothing. Two steps procedure is used to reduce small sample biasness (Leary and Michaely, 2011). Fist payout ratio is estimated by then target payout ratio (TPR) is calculated as firm's median payout ratio for the entire sample period. Deviation from the target dividend for each firm-year observation is estimated through;


(2) $$\begin{array}{l} \text{Δ}{\text{D}}_{it}={\text{β}}_{0}+{\text{β}}_{1}\;Dev_{it}+{\text{ε}}_{it} \end{array}$$

Where Dev_*it*_ = TPR_*i*_ × EPS_*it*_− *DPS*_*it*− 1_

Here the speed of adjustment (SOA) is represented by β_1_ estimated over the rolling window of 6 years for each firm-year observation.

In the case of relative volatility first, scaled earnings are estimated by multiplying earnings per share for removing the scaled effect. Then following Leary and Michaely (2011) quadratic time trend is fitted to both dividend per share and scaled earnings.


(3) $$\begin{array}{l} DPS_{it}={\text{β}}_{0}+{\text{β}}_{1}\;t+{\text{β}}_{2}{\text{t}}^{2}+{\text{U}}_{it} \end{array}$$

(4) $$\begin{array}{l} {\text{TPR}}_{i}\times {\text{EPS}}_{it}={\text{β}}_{0}+{\text{β}}_{1}\;t+{\text{β}}_{2}{\text{t}}^{2}+{\text{V}}_{it} \end{array}$$

Controlling for the linear trend reports the same level of dividend smoothing for two firms with targeting specific DPS and specific changes in DPS. While square time trend inclusion reports the same level of dividend smoothing for firms targeting the same percentage change in dividends. equation (3) and (4) estimates for each firm and then their relative volatility (second measure of dividend smoothing) is measured from the standard deviations of error terms over 6 years rolling window, given by


$$\begin{array}{l} \text{Relative volatility}=\text{σ}\left({\text{u}}_{\text{i},\text{t}}\right)/\text{σ}\left({\text{v}}_{\text{i},\text{t}}\right) \end{array}$$

### Ownership structure

The independent variables in the study are related to the ownership structure of the firms. The study has incorporated ownership concentration, individual ownership, foreign ownership, institutional ownership, management ownership and family ownership as types of ownerships in the study, described as under.

### Research model

#### Ownership structure and dividend smoothing

The impact of ownership on dividend smoothing is examined *via* the following empirical model.


(5) $$\begin{array}{l} SOA={\text{β}}_{0}+{\text{β}}_{1}\text{X}+{\text{β}}_{2}Top5\_own+{\text{β}}_{3}Indiv+{\text{β}}_{4}Foreign\\ \;+{\text{β}}_{5}Inst+{\text{β}}_{6}Mgt+{\text{ε}}_{it} \end{array}$$

(6) $$\begin{array}{l} Rel\_Vol={\text{β}}_{0}+{\text{β}}_{1}\text{X}+{\text{β}}_{2}Top5\_own+{\text{β}}_{3}Indiv+{\text{β}}_{4}Foreign\\ \;+{\text{β}}_{5}Inst+{\text{β}}_{6}Mgt+{\text{ε}}_{it} \end{array}$$

Then ownership index (Own_Index) is developed through principal component analysis (PCA) and its impact is estimated on dividend smoothing.


(7) $$\begin{array}{l} SOA={\text{β}}_{0}+{\text{β}}_{1}\;Own_{Index}+{\text{ε}}_{it} \end{array}$$

(8) $$\begin{array}{l} Rel\text{\_}Vol={\text{β}}_{0}+{\text{β}}_{1}\;Own_{Index}+{\text{ε}}_{it} \end{array}$$

Here X represents a vector of control variables.

#### Asymmetric dividend smoothing

Market reaction to the increases and cuts is not systematic. Therefore, the deviations are classified into two classes in the following pattern.


(9) $$\begin{array}{l} Dev_{P}=Dev\;if\;Dev\geq 0\text{ and otherwise zero}\\ Dev_{N}=Dev\;if\;Dev<0\;and\;otherwise\;zero\\ \text{ Δ}{\text{D}}_{it}={\text{β}}_{0}+{\text{β}}_{1}\;Dev_{P}+{\text{β}}_{2}\;Dev_{N}+{\text{ε}}_{it} \end{array}$$

Equation (9) investigates asymmetry of dividend smoothing, i.e., whether β_1_ and β_2_ are different. The null hypothesis for the above model is β_1_ = 0, β_2_ = 0. Both β_1_ and β_2_ need to be positive in order to show convergence toward the target payout ratio. Consequently, it is expected that the benefit of announcing an increase in dividends is lower than the cost of dividends cuts so managers may be more reluctant to cuts rather than announce increases. They are expected to adjust more quickly if they are below the target dividend than when they are adjusting downwards. Ultimately 0< β_2_ < β_1_ is expected.

#### Frequency of dividend changing events in Pakistan

Following Chemmanur et al. (2010) dividend events are classified into increases, cuts, continuations, dividend initiations, and dividend omissions in the following manner, as reported in Table 2.

**Table 2:** Definitions of dividend events.

**S. No**	**Event**	**Definition**
1	Increase	1 if Dividend per share in a firm-year has increased by more than 10%, 0 otherwise
2	Cut	1 if Dividend per share in a firm-year has decreased by more than 10%, 0 otherwise
3	Continuation	1 for any increase and decrease < 10%, 0 otherwise
4	Initiation	1 if a firm has announced dividend this year and didn't announce in lag period, 0 otherwise
5	Omission	1 if a firm has omitted dividend current year and announced in the lag period, 0 otherwise
6	Other	1 for all events other than initiations and omissions, 0 otherwise

Source: Author's calculation (2017).

## Results and discussion

### Descriptive statistics

Table 3 has listed descriptive statistics for both measures of dividend smoothing. Speed of adjustment (SOA) is measured with the help of Lintner (1956), modified by Fama and Babiak (1968). Both SOA and relative volatility are calculated for 6 years rolling window for each firm-year observation. The mean value for SOA and relative volatility were 0.488 and 1.442 consecutively.

**Table 3:** Descriptive statistics.

**Variable**	**Obs**	**Mean**	**Std. Dev**.	**Min**	**Max**
**Dividend smoothing**					
SOA	2,624	0.4886754	0.5195952	−0.8915795	2.268594
Relative-volatility	2,704	1.442002	1.880919	0.0062984	11.20815
**Ownership**	
Top5_own	1,442	0.6422336	0.2012416	0.004421	0.9995117
Mgt_own	1,467	0.2206203	0.2582157	0	0.9570594
Inst_own	1,471	0.1376284	0.1501357	0	0.9395854
Foreign_own	1,458	0.0603485	0.1711186	0	0.9886239
Family_own	1,453	0.735031	0.4414687	0	1
Indiv_own	1,464	0.211291	0.1813603	0	0.9991487

Source: Author's calculation (2017).

### Dividend payers *vs*. non-payers

The first column of Table 4 displays means values of the firm-level characteristic of the non-payers (firms that never paid a dividend during 1999–2015) while the second column displays mean values for payers. The last column displays the significance of the mean difference. Two-tail *t*-tests for unequal distributions are used for testing means. Table depicts non-dividend-paying with higher ownership concentration therefore are exposed to lower level of Type-I agency conflict. Similarly, in line with agency theory dividend-paying firms are with higher institutional ownership as compared to non-dividend-paying firms. Table also depicts consistent with the rental hypothesis dividend-paying firms with a higher proportion of foreign ownership. Sample also depicts that family firms pay more dividends. It might be because they want to signal to the market for decreasing chances of expropriation in family firms.

**Table 4:** Dividend payer vs. non-payers.

**Variable**	**Non-payer**	**Payer**	**(Non)-(Payer)**	**Sign**	**(*p*)**
Size	13.64681	14.76147	−1.114662	0.0000	^***^
Cash flow	10.67057	12.24106	−1.570492	0.0000	^***^
Leverage	2.045772	0.6468245	1.398948	0.1240	
Tangibility	0.633798	0.5339797	0.0998183	0.0000	^***^
Beta	−0.0477156	0.5051824	−0.5528979	0.6196	
Age	3.189276	3.359826	−0.2026349	0.0000	^***^
Turnover	12.2432	11.97213	0.2710741	0.0009	^***^
MORE	0.1997076	1.116363	−0.9166555	0.0946	^*^
Indexed	0.0195918	0.1150925	−0.0955006	0.0000	^***^
Earnings_Vol	2.954165	13.89136	−10.9372	0.0028	^***^
Slack	−2.343734	0.0997403	−2.443474	0.0948	^*^
**Ownership structure**				
Top5_own	0.6612695	0.6453278	0.0159416	0.3951	
Block1	0.3810545	0.3572798	0.0237747	0.0822	^*^
Mgt_own	0.2458335	0.2337116	0.0121219	0.5273	
Inst_own	0.0942655	0.137085	−0.0428195	0.0007	^***^
Foreign_own	0.0204119	0.0583734	−0.0379614	0.0000	^***^
Family_own	0.6607143	0.7318527	−0.0711384	0.0631	^*^
Indiv_own	0.2463516	0.2753338	−0.0289822	0.4309	

* p < 0.05,

** p < 0.01,

*** p < 0.001.

### Smoothing and non- smoothing firms

Both panels of Table 5 depict consistently with information asymmetry theory that firms that smooth more (based on SOA and relative volatility) are small in size. Small firms usually have more information asymmetry because of more volatile earnings, less coverage by media, and less following by analysts, therefore small firms smooth more. Similarly, the study also finds that firms with small cash flow smoothly more which is consistent with the financial constraints view of dividend smoothing, where high dividend smoothing is associated with low dividends. Therefore, firms having low cash flows are reluctant to announce an increase in dividends because of precautionary motives. Both SOA and relative volatility show that firms with high leverage and high tangibility exhibit more dividend smoothing.

**Table 5:** Firm characteristics across dividend smoothing quintiles.

**Panel A:**	**SOA quintile**						
	**1**	**2**	**3**	**4**	**5**	**Mean (1-5)**	**Significance**
**Variable**							
Size	14.82948	14.99703	15.09013	15.30918	15.04418	−0.2147061	0.0369
Cash flow	0.0503799	0.0664784	0.0688423	0.0760415	0.0859161	−0.0355362	0.0000
Leverage	0.6491198	0.5788076	0.5509034	0.5423628	0.5486662	0.1004536	0.0000
Tangibility	0.5099051	0.4890019	0.490463	0.4862442	0.4915393	0.0183658	0.2095
Beta	0.5945517	0.4677498	0.4142299	0.494975	0.4697985	0.1247532	0.0018
Age	3.458493	3.489264	3.444815	3.492499	3.370525	0.0879685	0.0058
Turnover	11.96952	11.65531	11.62336	12.21667	11.88452	0.0849995	0.6667
**Ownership structure**
Top5_own	0.658032	0.6358552	0.6202901	0.6185526	0.6757265	−0.0176946	0.3159
Mgt_own	0.2309293	0.2197441	0.2690948	0.2020844	0.1921724	0.0387568	0.0885
Inst_own	0.1443721	0.1426811	0.1382664	0.1418825	0.1278788	0.0164933	0.1938
Foreign_own	0.0456935	0.0621722	0.0462523	0.0692012	0.0847516	−0.0390581	0.0201
Indiv_own	0.2192274	0.2169783	0.2179457	0.2229376	0.1988428	0.0203846	0.2081
**Panel B:**	**Relative volatility quintile**						
	**1**	**2**	**3**	**4**	**5**	**Mean(1–5)**	**Significance**
**Variable**							
Size	14.67578	15.03134	15.21502	15.17769	15.15158	−0.4758003	0.0000
Cash flow	0.0385599	0.0724445	0.0647047	0.076296	0.0917185	−0.0531586	0.0000
Leverage	0.6845038	0.559124	0.5574618	0.5490562	0.5279767	0.1565272	0.0000
Tangibility	0.5223481	0.4957681	0.4818158	0.4991358	0.4728248	0.0495234	0.0003
Beta	0.5308417	0.4550994	0.5137163	0.485049	0.490659	0.0401827	0.2822
Age	3.446287	3.494553	3.497874	3.42962	3.326298	0.1199898	0.0001
Turnover	11.61117	11.85876	12.21737	12.01765	11.97097	−0.3598046	0.0671
**Ownership structure**
Top5_own	0.6177073	0.6150351	0.6472949	0.6436648	0.6808097	−0.0631024	0.0004
Mgt_own	0.2815171	0.248691	0.2104248	0.1925158	0.1809065	0.1006105	0.0000
Inst_own	0.1452608	0.1491803	0.1553811	0.1254728	0.1149883	0.0302725	0.0103
Foreign_own	0.0126103	0.0434779	0.0489977	0.0725784	0.0725784	0.1091438	0.0000
Indiv_own	0.2375641	0.2255531	0.2048111	0.229996	0.1791065	0.0584576	0.0001

Pakistan corporate ownership is structured by a family-concentrated structure. Consistent with agency theory and in support of H_1_. Firms smoothing more are having less level of ownership concentration. Similarly, the study found firms with high institutional ownership to be associated with a high level of dividend smoothing as institutional owners are efficient in monitoring. Firms with more individual owners exhibit more dividend smoothing as they are subject to more information asymmetry as they deal with more informed investors. Higher dividend smoothing is associated with a higher level of ownership by the directors and their spouses and children, which might be because of the rent-seeking and risk-aversive behavior of the managers. The same association is also found in the case of foreign ownership.

### Ownership structure and dividend smoothing

Table 6 depicts the effect of ownership structure on dividend smoothing while controlling for the risk, growth opportunities, size, leverage, and firm tangibility. First column of the table has used the speed of adjustment as the dependent variable and is the result of Tobit regression while second column has relative volatility as the dependent measure.

**Table 6:** Ownership structure and dividend smoothing.

**VARIABLES**	**SOA**	**Rel_Vol**
Indiv_own	−0.0698^***^	−0.269^**^
	(0.0221)	(0.118)
Foreign_own	0.0868^***^	0.152
	(0.0279)	(0.141)
Inst_own	−0.0367^*^	−0.561^***^
	(0.0205)	(0.109)
Mgt_own	−0.116^***^	−0.507^**^
	(0.0445)	(0.244)
Top5_own	0.3354^*^	0.629^***^
	(0.1715)	(0.184)
MBR	0.00961^**^	0.0779^***^
	(0.00465)	(0.0235)
Beta	−0.0235	−0.260^*^
	(0.0221)	(0.144)
Size	−0.0272^***^	−0.0253
	(0.00858)	(0.0398)
Leverage	0.0900^**^	−0.694^***^
	(0.0442)	(0.269)
Tangibility	0.0429	0.354
	(0.0586)	(0.276)
Constant	0.849^***^	1.939^***^
	(0.137)	(0.651)
Observations	1,214	995
R-squared		0.075
F-Stat		7.95
Prob > firm		0.0000
Left-censored	77	
Right-censored	126	
LR chi2	255.36	
Prob > chi2	0.0000	
Pseudo R2	0.1994	
Log-likelihood	−522.684	
Industry FE	Yes	Yes

Standard errors in parentheses.

*** p<0.01,

** p<0.05,

* p<0.1.

Consistent with H_2_ individual ownership is associated negatively with dividend smoothing in case of both dividend smoothing measures. Firms crowded by individual owners are facing high levels of agency conflict and are subject to severe information asymmetry being uninformed investors. Therefore, they substitute their higher information asymmetry and risk of being exploited by insiders *via* demanding large and smooth dividends. This result is consistent with agency theory of dividend smoothing and is in line to the studies by Brennan and Thakor (1990) and Jeong (2013).

Firms having more foreign ownership opt for less dividend smoothing. Consistent to rental hypothesis in foreign-owned firms, dividends will not stick to the previous level but will be frequently changed with variation in earnings as these firms are less constrained by financial resources. Also such firms are less constraint (global advantage hypothesis) and foreign owners are efficient monitors so parallel to Park and Chung (2007), these results are consistent with external constraints as well as with information asymmetric channel. The authors could not find significant result for foreign ownership in case of relative volatility. parallel to Leary and Michaely (2011) and Javakhadze et al. (2014) institutional ownership is positively associated with dividend smoothing. The preference of intuitional owners for high and steady dividends and their ability of reacting for cuts and omissions ensures high dividend smoothing. Parallel to Bradford et al. (2013) management ownership and dividend smoothing are positively associated. This result is consistent with the signaling theory of dividends as firms with low management ownership are exposed to severe agency conflict which is reduced *via* alternative monitoring mechanism of paying high dividends (La Porta et al., 2000). So they signal to market through large and stable dividends. Also these results are consistent with rent extraction behavior *via* opting higher level of dividend smoothing.

Ownership concentration is negatively associated with dividend smoothing in parallel to Leary and Michaely (2011), Jeong (2013) and Javakhadze et al. (2014). It suggests that firms with concentrated ownership are higher alignment and have lower level of agency conflict. Therefore they are less concernced with dividend cuts and omissions rather they are more concern with long term survival of the firm. The results are parallel to Ali et al. (2021).

### Ownership index and dividend smoothing

Following Durana et al. (2022b), this study uses principal component analysis (PCA) for developing ownership index (Own_index_). The main purpose of PCA is to reduce the number of variables into uncorrelated components (Durana et al., 2022b). Table 7 depicts weights of all the mentioned five variables in the ownership index for Pakistani firms during the sample period based on principal components analysis.

**Table 7:** Weights of the ownership index.

**Variables**	**Own_Index_**
Foreign_own	0.3998
Inst_own	0.2302
Mgt_own	−0.5169
Top5share	0.4834
Individual	−0.535
Kaiser-Meyer-Olkin Statistic	0.522
Bartlett's Chi-square	352.624
Bartlett's test *p*-value	0.0000

Source: Authors' calculation.

In Table 8, the authors have regressed the speed of adjustment and relative volatility with the ownership index developed *via* PCA by incorporating foreign ownership, institutional ownership, management ownership, and ownership concentration measured through proportionate ownership of the five largest shareholders and proportionate ownership owned by individuals.

**Table 8:** Ownership index and dividend smoothing.

**Variables**	**SOA**	**Rel_Vol**
Owner_index_	0.0462^***^	0.1433^*^
	(0.00962)	(0.0741)
MBR	0.0110^**^	0.0847^***^
	(0.00464)	(0.0243)
Beta	−0.0268	−0.318^**^
	(0.0217)	(0.146)
Size	−0.0225^***^	−0.0348
	(0.00823)	(0.0398)
Leverage	0.0828^*^	−0.957^***^
	(0.0442)	(0.279)
Tangibility	0.0401	0.262
	(0.0582)	(0.325)
Constant	0.749^***^	2.514^***^
	(0.121)	(0.596)
Observations	1,214	995
R-squared		0.102
F-Stat		6.55
Prob > firm		0.0000
Left-censored	77	
Right-censored	126	
LR chi2	243.87	
Prob > chi2	0.0000	
Pseudo R2	0.1904	
Log-likelihood	−518.42866	
Industry FE	Yes	Yes

Standard errors in parentheses.

*** p<0.01,

** p<0.05,

* p<0.1.

As depicted in the table, a negative association of ownership index and dividend smoothing is witnessed while using both proxies SOA and relative volatility, which is in line with the substitution hypothesis. Dividend smoothing mitigates agency conflict and reduces information asymmetry between insiders and outsiders, but the above regression shows that this relationship is affected by the ownership structure of the firm. These results are parallel to La Porta et al. (2000) and Javakhadze et al. (2014) which is that firms with more institutional and management and individual ownership will smooth more while firms with more ownership concentration and foreign ownership will smooth less. A high score on the ownership index represents firms with more institutional, more management, more individual ownership, and with less foreign and less concentrated ownership. Firms with efficient ownership will practice less dividend smoothing than firms with inefficient ownership.

### Ownership structure and asymmetric dividend smoothing

Table 9 depicts a positive and significant coefficient for the interaction term of foreign ownership with positive deviations (*Dev*_*P*_), it suggests that firms having more foreign ownership quickly adjust their dividends toward the target level when the managers are adjusting their dividends from below. It means that foreign ownership has a positive influence on the speed of adjustment from below which affirms hypothesis (H_3_) of the study.

**Table 9:** Ownership structure and asymmetric dividend smoothing.

**Variables**	**DPS_t_-DPS_t − 1_**	**DPS_t_-DPS_t − 1_**
*Dev_*P*_* * Foreign_own	4.207^***^	4.176^***^
	(0.789)	(0.799)
*Dev_*N*_* * Foreign_own	−0.361^*^	−0.433^**^
	(0.194)	(0.196)
*Dev_*P*_* * Inst_own	1.375	1.403
	(1.278)	(1.292)
*Dev_*N*_* * Inst_own	−1.921^***^	−2.096^***^
	(0.741)	(0.746)
*Dev_*P*_* * Mgt_own	−2.667^***^	−2.606^***^
	(0.931)	(0.937)
*Dev_*N*_* * Mgt_own	−2.240^**^	−2.313^**^
	(0.934)	(0.938)
*Dev_*P*_* * Top5_own	1.757^**^	1.897^**^
	(0.805)	(0.815)
*Dev_*N*_* * Top5_own	1.792^**^	1.734^**^
	(0.733)	(0.738)
*Dev_*P*_* * Indiv_own	0.939^***^	0.955^***^
	(0.260)	(0.263)
*Dev_*N*_* * Indiv_own	−0.536^***^	−0.525^***^
	(0.144)	(0.145)
Foreign_own	11.30^**^	13.34^**^
	(5.206)	(5.393)
Inst_own	−3.891	−4.411
	(5.350)	(5.411)
Mgt_own	−3.539	−2.784
	(4.503)	(4.579)
Top5_own	4.544	3.058
	(3.854)	(3.977)
Indiv_own	−0.538	−0.265
	(1.322)	(1.377)
*Dev_*P*_*	−0.458	−0.612
	(0.649)	(0.657)
*Dev_*N*_*	0.422	0.531
	(0.610)	(0.615)
F-Stat	67.25	41.36
Prob > firm	0.0000	0.0000
Industry Effect	No	Yes

Standard errors in parentheses.

*** p<0.01,

** p<0.05,

* p<0.1.

Table 9 depicts that impact of institutional ownership on dividend smoothing is not significant when a firm is adjusting from below toward the target dividend, however when a firm is adjusting its dividend from above, then the institutional ownership has negative impact. It suggests that institutional ownership is associated with a higher level of smoothing in this case, shows strong preference of institutional investors for large and stable dividends. It means that institutional investors resist cuts and omissions of dividends which is consistent with (H_4_) of the study. These results are consistent with agency theory and parallel to La Porta et al. (2000) and Javakhadze et al. (2014).

No matter whether the firm is below the target dividend or above, the impact of management ownership on speed of adjustment is negative. It suggests that management ownership and dividend smoothing are positively associated in both cases whether adjusting dividends from below or above. They smooth more when smoothing from below as they consider high dividends as commitments and smoothing from above is consistent with signaling hypothesis. These results are parallel to Leary and Michaely (2011) who claimed dividend smoothing, to be the result of a manager's persistent behavior.

Consistent with the agency and information asymmetric view firms with concentrated ownership can bear with dividends cuts and omissions, they smooth less and are more concerned with the long term survival of the firm which is parallel to Leary and Michaely (2011). Table depicts firms with high individual ownership impacts SOA positively when they are smoothing from below and its impact is negative when firms are adjusting from above. However, the results are not significant, which may be because of their inefficient influence as the corporate structures in Pakistan are dominated by family concentrated ownership.

Consistent with rent-seeking hypothesis foreign owners smooth less from below and smooth more from above i.e., they always prefer high dividends. The study finds that institutional investors increase smoothing from above only as they are more concerned about avoiding cuts and omissions. Consistent with the persistent behavior hypothesis (dividend smoothing is the outcome of managerial habit) they smooth in either case. Concentrated firms smooth less in either case however we couldn't observe the significant result in asymmetric analysis which may be because of their inefficient influence in the weak governing environment of Pakistan.

### Ownership structure and dividend changing events

The sample is divided into two sub groups based on the proportionate foreign ownership, such that observations having foreign ownership greater than the mean value of foreign ownership were categorized as firms with high foreign ownership while those below mean value are firms with low foreign ownership. Table 10A depicts that firms with high foreign ownership have 37.96% dividend increasing events against 30.75% by the firms with low foreign ownership. While they have 28.24% cuts against 28.47% by those with low foreign ownership. In Table 10B, firms with high foreign ownership have continuations of 33.8% against 40.77% by low foreign ownership firms. It shows that firms with high foreign ownership observes more dividend increasing events which is consistent with the rental hypothesis.

**Table 10A:** Owner structure and Frequency of dividend changing events.

**Panel: A**	**Increases**	**Continuations**	**Cuts**	**Total**
**Foreign Own**				
High	82	73	61	216
	37.96%	33.8%	28.24%	100%
Low	310	411	287	1,008
	30.75%	40.77%	28.47%	100%
Total	392	484	348	1,224
	32.03%	39.54%	28.43%	100%
Chi-square tests of independence	5.08			
(*p*-Value)	(0.079)			
**Inst_own**				
High	215	317	204	736
	29.21%	43.07%	27.72%	100%
Low	178	172	148	498
	35.74%	34.54%	29.72%	100%
Total	393	489	352	1,234
	31.85%	39.63%	28.53%	100%
Chi-square tests of independence	9.8522			
(*p*-Value)	(0.007)			
**Mgt_own**				
High	141	217	135	493
	28.6%	44.02%	27.38%	100%
Low	252	270	216	738
	34.15%	36.59%	29.27%	100%
Total	393	487	351	1,231
	31.93%	39.56%	28.51%	100%
Chi-square tests of independence	7.341			
(p-Value)	(0.025)			
**Top5_own**				
High	214	245	191	650
	32.92%	37.69%	29.38%	100%
Low	179	243	161	583
	30.7%	41.68%	27.62%	100%
Total	393	488	352	1,233
	31.87%	39.58%	28.55%	100%
Chi-square tests of independence	2.0474			
(*p*-Value)	(0.359)			
**Individual own**				
High	136	217	121	474
	28.69%	45.78%	25.53%	100%
Low	256	267	231	754
	33.95%	35.41%	30.64%	100%
Total	392	484	352	1,228
	31.92%	39.41%	28.66%	100
Chi-square tests of independence	11.0278			
(*p*-Value)	(0.004)			

Source: Author's calculation (2017).

**Table 10B:** Ownership structure and frequency of dividend changing events.

**Panel: B**	**Initiations**	**Omissions**	**Others**	**Total**
**Foreign own**				
High	13	18	155	186
	6.99%	9.68%	83.33%	100%
Low	112	90	586	788
	14.21%	11.42%	74.37%	100%
Total	125	108	741	974
	12.83%	11.09%	76.08%	100%
Chi-square tests of independence	8.1224			
(*p*-Value)	(0.017)			
**Inst_own**				
High	48	44	323	415
	11.57%	10.6%	77.83%	100%
Low	80	65	422	567
	14.11%	11.46%	74.43%	100%
Total	128	109	745	982
	13.03%	11.1%	75.87%	100%
Chi-square tests of independence	1.7152			
(*p*-Value)	(0.424)			
**Mgt_own**				
High	73	64	483	620
	11.77%	10.32%	77.9%	100%
Low	55	45	261	361
	15.24%	12.47%	72.3%	100%
Total	128	109	744	981
	13.05%	11.11%	75.84%	100%
Chi-square tests of independence	3.9825			
(*p*-Value)	(0.137)			
**Top5_own**				
High	69	53	334	456
	15.13%	11.62%	73.25%	100%
Low	59	56	411	526
	11.22%	10.65%	78.14%	100%
Total	128	109	745	982
	13.03%	11.1%	75.87%	100%
Chi-square tests of independence	3.852			
(*p*-Value)	(0.146)			
**Indiv_own**				
High	65	66	501	632
	10.28%	10.44%	79.27%	100%
Low	63	43	243	349
	18.05%	12.32%	69.63%	100%
Total	128	109	744	981
	13.05%	11.11%	75.84%	100%
Chi-square tests of independence	13.866			
(*p*-Value)	(0.001)			

Source: Author's calculation (2017).

Firms with high institutional ownership have more continuations (43%) than firms with low institutional ownership (34.5%). Shows their monitoring behavior. After dividing the sample into high and low management ownership sub samples, the authors found high management ownership firms have more continuations and fewer increases and cuts than the low management groups.

Table 10B depicts firms with high institutional ownership in Pakistan reports fewer initiations and omissions against their counterparts. This suggests a higher level of dividend smoothing and less dividend changing events like dividend initiations and omissions are associated with a high level of institutional ownership. These results affirm hypothesis (H_4_) of the study. These results show institutional owners' preference for stable dividends. The results are parallel La Porta et al. (2000) and Javakhadze et al. (2014).

Firms with high management ownership have fewer initiations and omissions which affirms hypothesis (H_5_) of the study. These results suggest that high management ownership is associated with high dividend smoothing.

Table 10B depicts that firms with high individual ownership have lower number of dividend initiations and omissions as compared to the firms with low individual ownership. These results suggest that firms with high individual ownership choose for a higher degree of dividend smoothing as reflected by dividend increases and cuts which affirms hypothesis (H_2_) of the study. These results are consistent with both agency and information asymmetry theory of dividend smoothing as individual owners are comparatively less informed.

### Endogeneity

This study investigates the impact of ownership structure on dividend smoothing. Even though found strong evidence that dividend smoothing is related to the ownership structure of the firm but in order to ensure that the results are not affected because of the endogeneity, the author has followed Kumar (2006). And have verified through the following regressions that the results are not affected by endogeneity. Both panels of Table 11 depict statistically insignificant coefficients for both measures of dividend smoothing (SOA and Rel_Vol) and its lag terms in the case of all the regressions tabulated in Table 11. As a statistically significant coefficient was not found for any of the lag terms of speed of adjustment and relative volatility in the case of all ownership variables, therefore it shows that results of ownership structure association with dividend smoothing of the study are not affected by endogeneity (Kumar, 2006).

**Table 11:** Dividend smoothing and ownership structure.

**Variables**	**Foreign_own**	**Inst_own**	**Mgt_own**	**Top5_own**	**Indiv_own**
**Panel: A**					
SOA	0.0102	−0.00471	0.00544	−0.00690	0.0116
	(0.0123)	(0.0101)	(0.0179)	(0.0193)	(0.0127)
SOA _t − 1_	−0.00816	0.00242	0.00297	0.0221	0.0102
	(0.0135)	(0.0110)	(0.0195)	(0.0211)	(0.0138)
SOA _t − 2_	−0.00601	−0.0161	0.0201	0.00622	0.00466
	(0.0120)	(0.00989)	(0.0175)	(0.0189)	(0.0124)
Constant	0.0662^***^	0.149^***^	0.202^***^	0.622^***^	0.194^***^
	(0.00933)	(0.00766)	(0.0136)	(0.0147)	(0.00962)
Observations	1,121	1,130	1,128	1,130	1,127
R-squared	0.001	0.004	0.003	0.002	0.004
**Variables**	**Foreign_own**	**Inst_own**	**Mgt_own**	**Top5_own**	**Indiv_own**
**Panel: B**					
Rel_Vol	2.87e−05	−0.000305	−0.000321	0.000616	−0.000164
	(0.000321)	(0.000261)	(0.000432)	(0.000429)	(0.000319)
Rel_Vol _t − 1_	2.24e−05	−0.000122	−9.99e−05	0.000255	−5.76e−05
	(0.000381)	(0.000309)	(0.000512)	(0.000508)	(0.000378)
Rel_Vol _t − 2_	−0.000124	−0.000318	−0.000372	0.000539	−0.000184
	(0.000320)	(0.000260)	(0.000431)	(0.000427)	(0.000317)
Constant	0.0677^***^	0.143^***^	0.202^***^	0.631^***^	0.203^***^
	(0.00569)	(0.00460)	(0.00763)	(0.00757)	(0.00563)
Observations	1,062	1,070	1,068	1,070	1,068
R-squared	0.000	0.009	0.004	0.012	0.002

Standard errors in parentheses.

*** *p*<0.01,

** *p*<0.05,

* *p*<0.1.

## Conclusion

Firms smooth dividends mainly for mitigating agency conflict or reducing information asymmetry. The ownership structure of the firm affects agency conflict as well as on information asymmetry. The study examines the impact of ownership structure on dividend smoothing in Pakistan. It answers questions like, how a company's ownership structure links to dividend smoothing. Does ownership behave differently when firms smooth from below and above the targeted payout ratio? To answers these, the sample of all non-financial listed on Pakistan stock exchange for the study period 2005–2015 was undertaken for both parametric and non-parametric analysis.

The study concluded that ownership concentration is negatively associated with dividend smoothing and the effect remains irrespective of smoothing from above or below. High dividend smoothing (*via* dividends increases and cuts) is practiced by low concentrated firms. Also, low ownership concentration is associated with a high frequency of dividend initiations and omissions. This result is consistent with the studies by Brennan and Thakor (1990) and Jeong (2013). Consistent with the rental hypothesis of dividends foreign owners opt for less dividend smoothing. While opposite impact of foreign ownership on SOA was observed when a firm is adjusting from below and above, they prefer high dividends. The relationship holds during non-parametric analysis as well. It means that in Pakistan, foreign investors, are less concerned about the stability of dividends as these firms are less constrained but want to recover their investment at the earliest. Institutional investors are positively associated with dividend smoothing. Asymmetric analysis of dividend smoothing revealed that institutional owners avoid cuts and omissions. It is because of the strong preference of institutional investors for large and stable dividends. Consistent with the information asymmetry theory positive association between dividend smoothing and ownership by management, their spouses and children are observed. They follow their habit of consistence *via* both smoothing from above and below.

The ownership index developed *via* incorporating foreign, institutional, management ownership, ownership concentration, individuals and family ownership, found to be negatively associated with dividend smoothing. High score on ownership index represents, firms with more institutional, more management, more individual ownership and with less foreign and less concentrated ownership.

Dividend smoothing mitigates agency conflict and reduces information asymmetry between insiders and outsiders. Consistentwith La Porta et al. (2000) and Javakhadze et al. (2014), the study revealed that this relationship is affected by the ownership structure of the firm. we also found consistent that family firms smooth more to win the trust of minor shareholders and to give signals that they are sacrificing their private benefits to reduce the type II agency problem, i.e., reducing chances of minor shareholders' expropriation. Being singly country analysis, the results may generalized to other developing countries with causation after considering legal and culture context. SOA is measured *via* 6 years' window, it may be lengthening in the future studies. Investor protection and disclosure quality could be potential determinants of dividend smoothing for future. SECP and PSX want to insure minority protection, the current findings reflect that dividend smoothing is another monitoring mechanism. Over all the findings of the current study provides insight to the investors and regulators by offering dividend smoothing as alternative monitoring mechanism to corporate governance.

## Data availability statement

The raw data supporting the conclusions of this article will be made available by the authors, without undue reservation.

## Author contributions

Supervised by WZ and YH. Idea write up and ananlysis by ZA. Reviewed and structured by SA. All authors contributed to the article and approved the submitted version.

## Funding

We acknowledge the support of Research on the Long-Term Mechanism and Anti-Relative Poverty based on Property Rights protection, supported by the National Social Science Foundation of China (Grant No. 21FGLB087).

## Conflict of interest

The authors declare that the research was conducted in the absence of any commercial or financial relationships that could be construed as a potential conflict of interest.

## Publisher's note

All claims expressed in this article are solely those of the authors and do not necessarily represent those of their affiliated organizations, or those of the publisher, the editors and the reviewers. Any product that may be evaluated in this article, or claim that may be made by its manufacturer, is not guaranteed or endorsed by the publisher.
